# Supplementary material for: Associations of habitual physical activity and carotid-femoral pulse wave velocity; a systematic review and meta-analysis of observational studies
Source: PLoS One. 2023 Apr 6;18(4):e0284164. doi: 10.1371/journal.pone.0284164 (PMC10079053; doi:10.1371/journal.pone.0284164)
Supplement: S1 File — (PDF) [file pone.0284164.s001.pdf]

S1 File. Base search strategy on MEDLINE database.

Ovid Search (MEDLINE):

1. ((physical* or habitual* or occupational) adj2 activ*).ti,ab.

2. (sedentary adj2 (activ* or time)).ti,ab.

3. (leisure adj2 time adj2 activ*).ti,ab.

4. (moderate adj2 vigorous adj2 activ*).ti,ab.

5. MVPA.ti,ab.

6. activ* time.ti,ab.

7. acceleromet*.ti,ab.

8. 1 or 2 or 3 or 4 or 5 or 6 or 7

9. ((vascula* or arter*) adj2 (function or health or stiff* or age* or disease or compliance)).ti,ab.

10. (endotheli* adj2 function).ti,ab.

11. (vasodilation or vasodilatation).ti,ab.

12. ((microvascular or macrovascular) adj2 function).ti,ab.

13. 9 or 10 or 11 or 12 or 13

14. (pulse adj2 velocity).ti,ab.

15. PWV.ti,ab.

16. 8 and (14 or 15)
