# Supplementary figures and images for: Associations of habitual physical activity and carotid-femoral pulse wave velocity; a systematic review and meta-analysis of observational studies
Source: PLoS One. 2023 Apr 6;18(4):e0284164. doi: 10.1371/journal.pone.0284164 (PMC10079053; doi:10.1371/journal.pone.0284164)

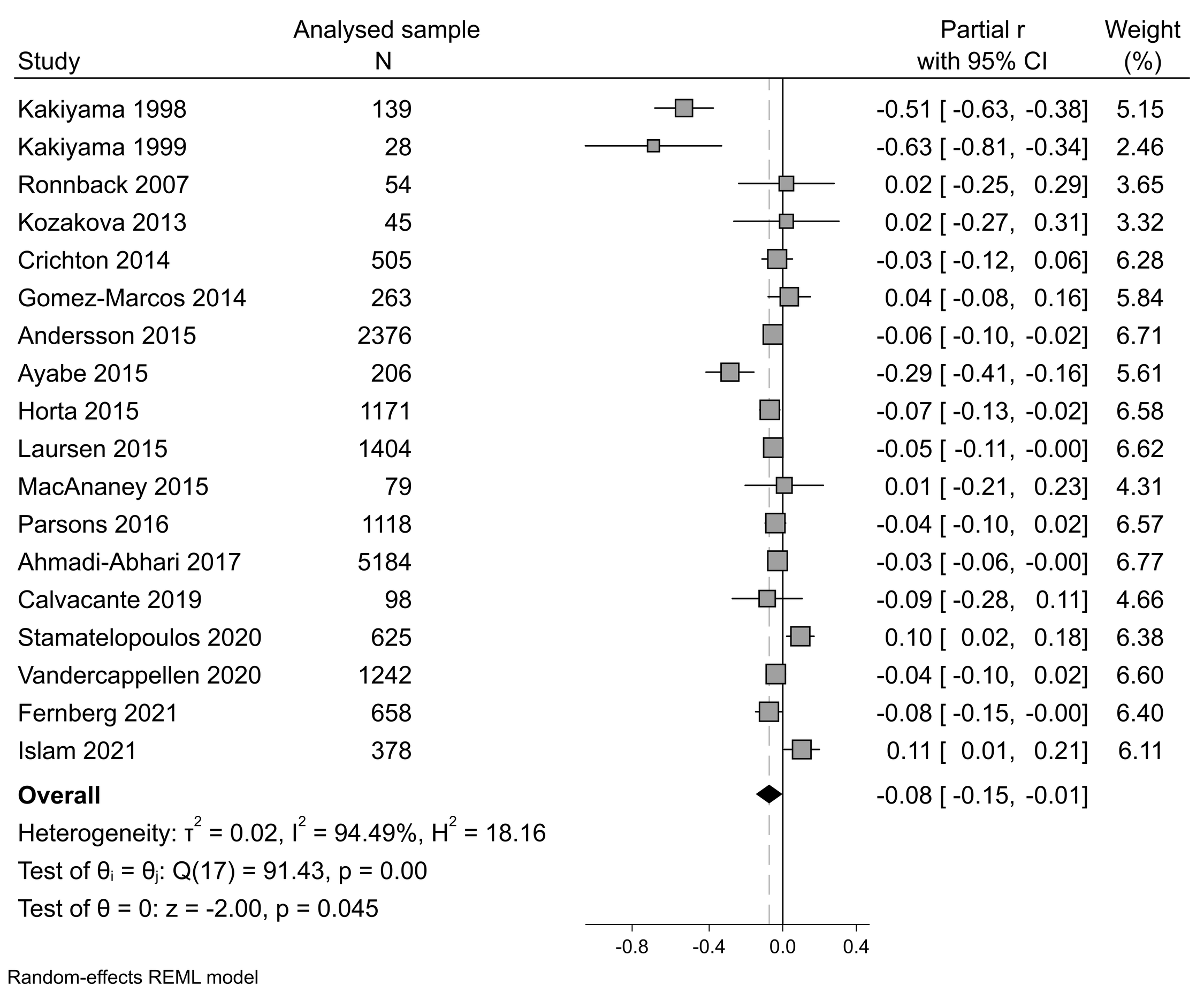

Supplement: S1 Fig — Random effects model. Grey squares indicate individual study association with 95% confidence intervals (CI). Solid vertical line represents line of no difference. Dashed vertical line and solid black diamond represents the overall summary estimate of association. (TIF) [file pone.0284164.s002.tif]

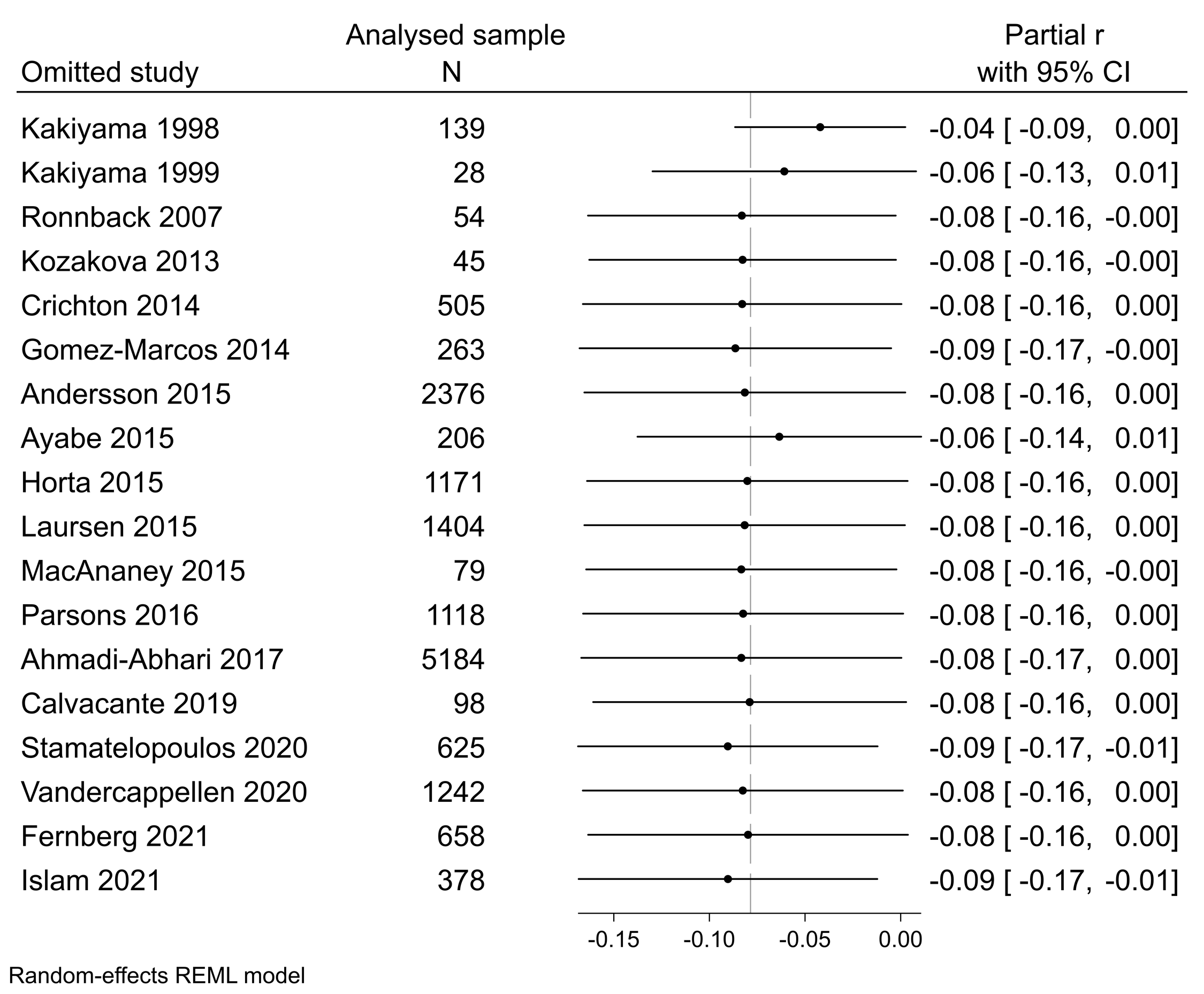

Supplement: S2 Fig — Grey dashed line indicates the overall effect size with all included studies (N = 18) as per main forest plot. Black circles indicate the resultant effect size (N = 17) when the current study is removed from the meta-analysis, with 95% confidence intervals. The overall association was not significantly changed by the removal of any one study. (TIF) [file pone.0284164.s003.tif]

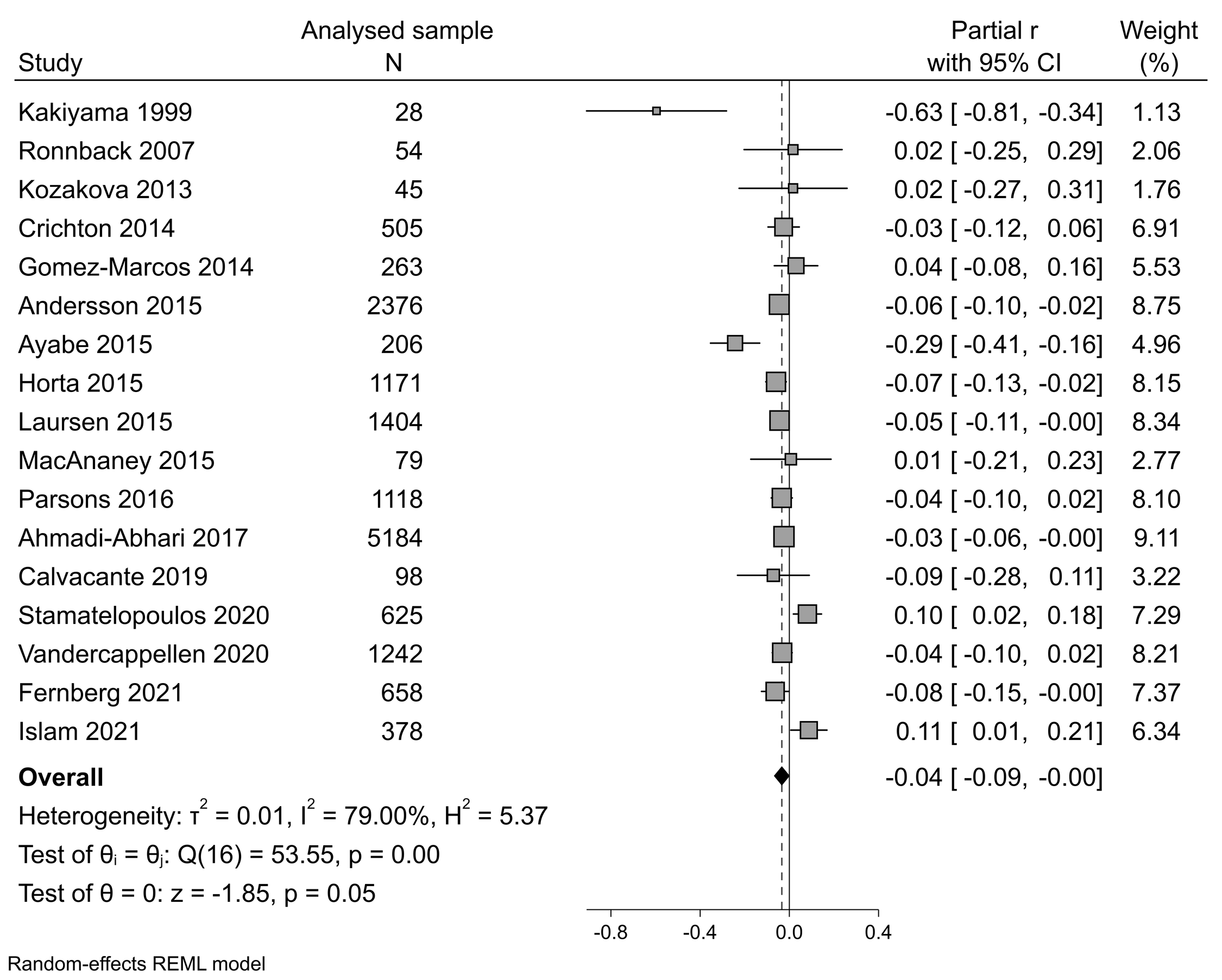

Supplement: S3 Fig — Random effects model. Grey squares indicate individual study association with 95% confidence intervals (CI). Solid vertical line represents line of no difference. Dashed vertical line and solid black diamond represents the overall summary estimate of association. (TIF) [file pone.0284164.s004.tif]

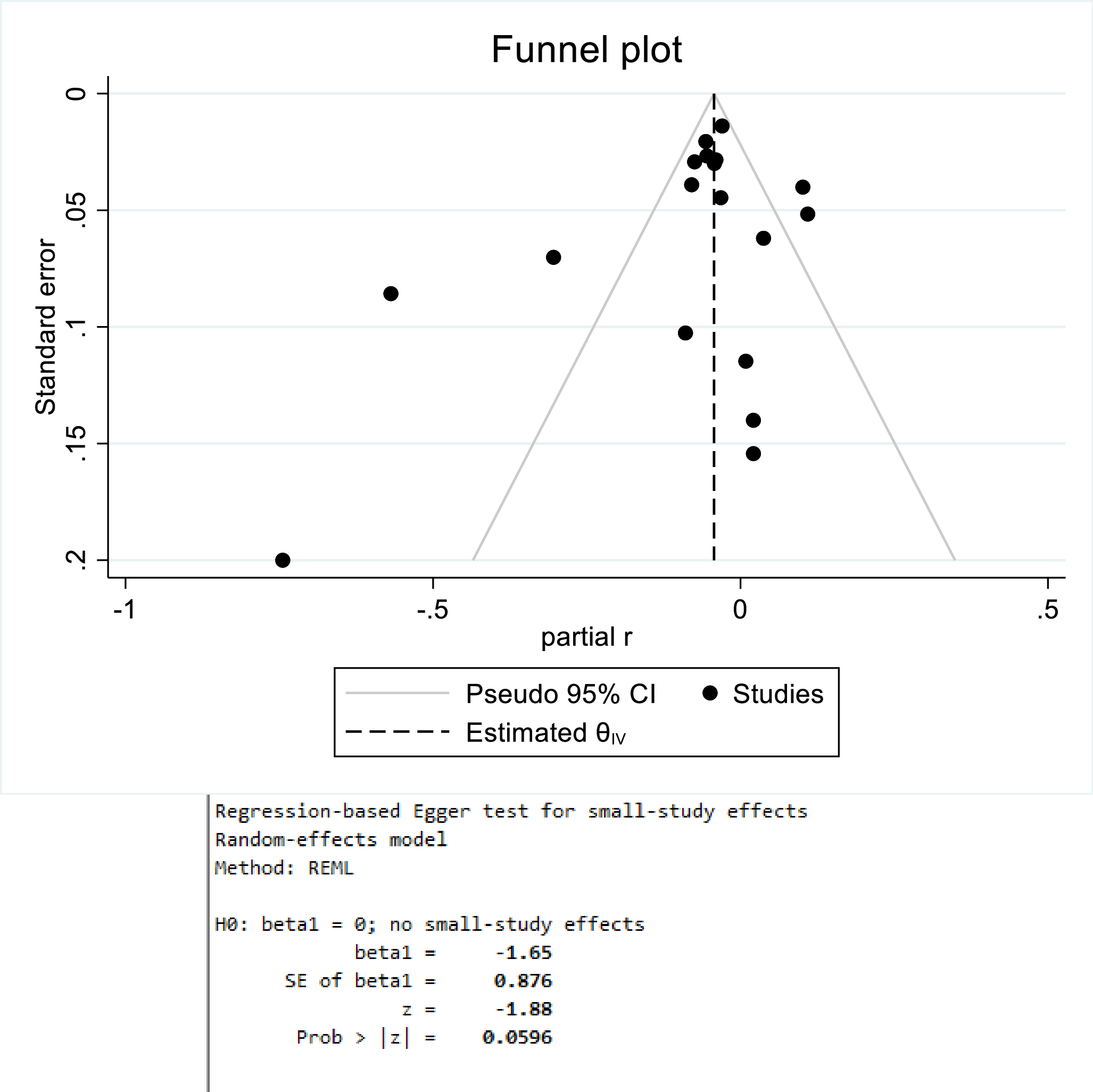

Supplement: S4 Fig — No small study effects were found. (TIF) [file pone.0284164.s005.tif]

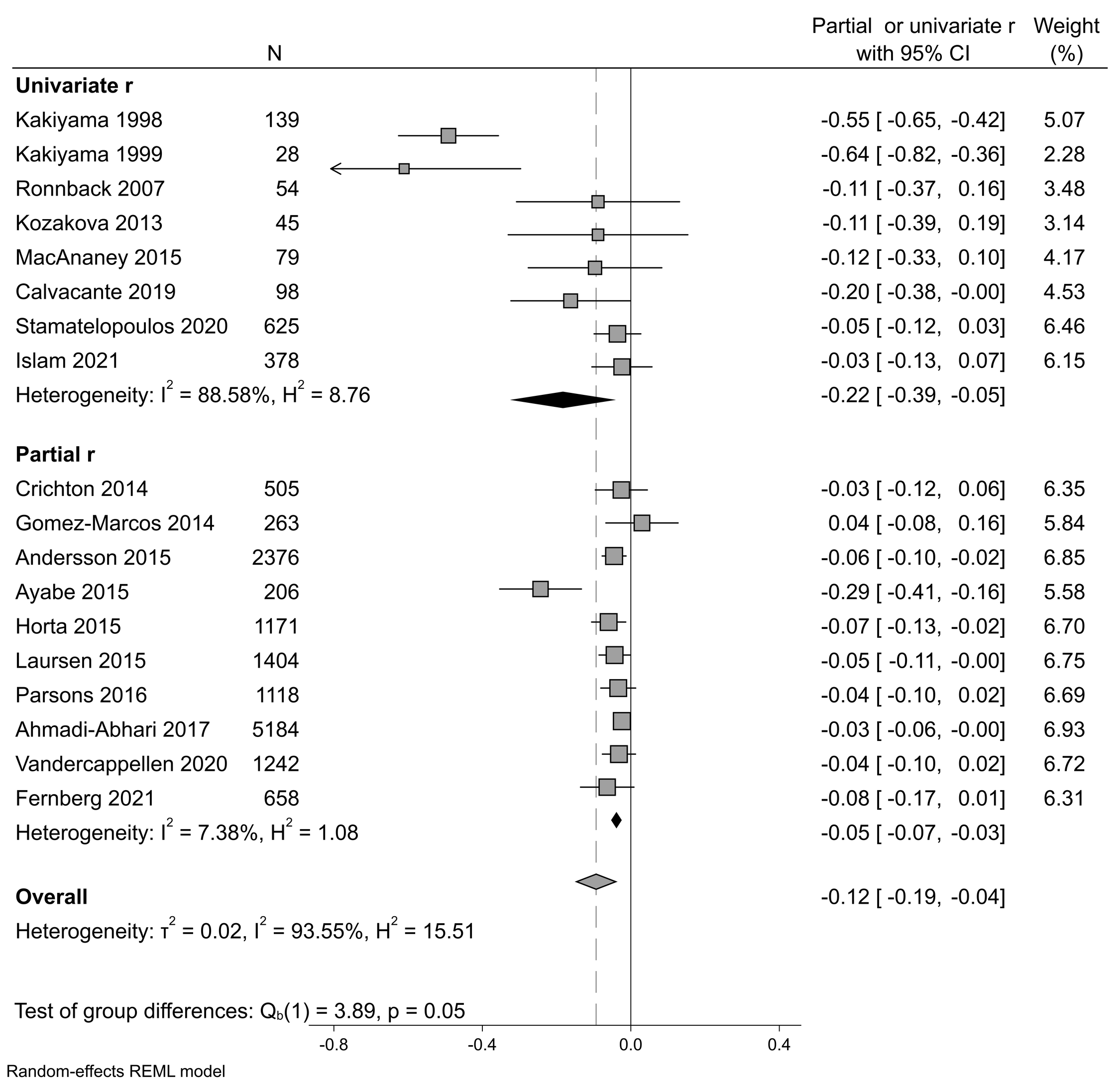

Supplement: S5 Fig — Top indicates univariate r as reported in publication and bottom indicates partial r adjusted for any number of covariates as reported in the publication. Random effects model. Grey squares indicate individual study association with 95% confidence intervals (CI). Solid vertical line represents line of no difference. Dashed vertical line and grey diamond represents the overall summary estimate of association. (TIF) [file pone.0284164.s006.tif]

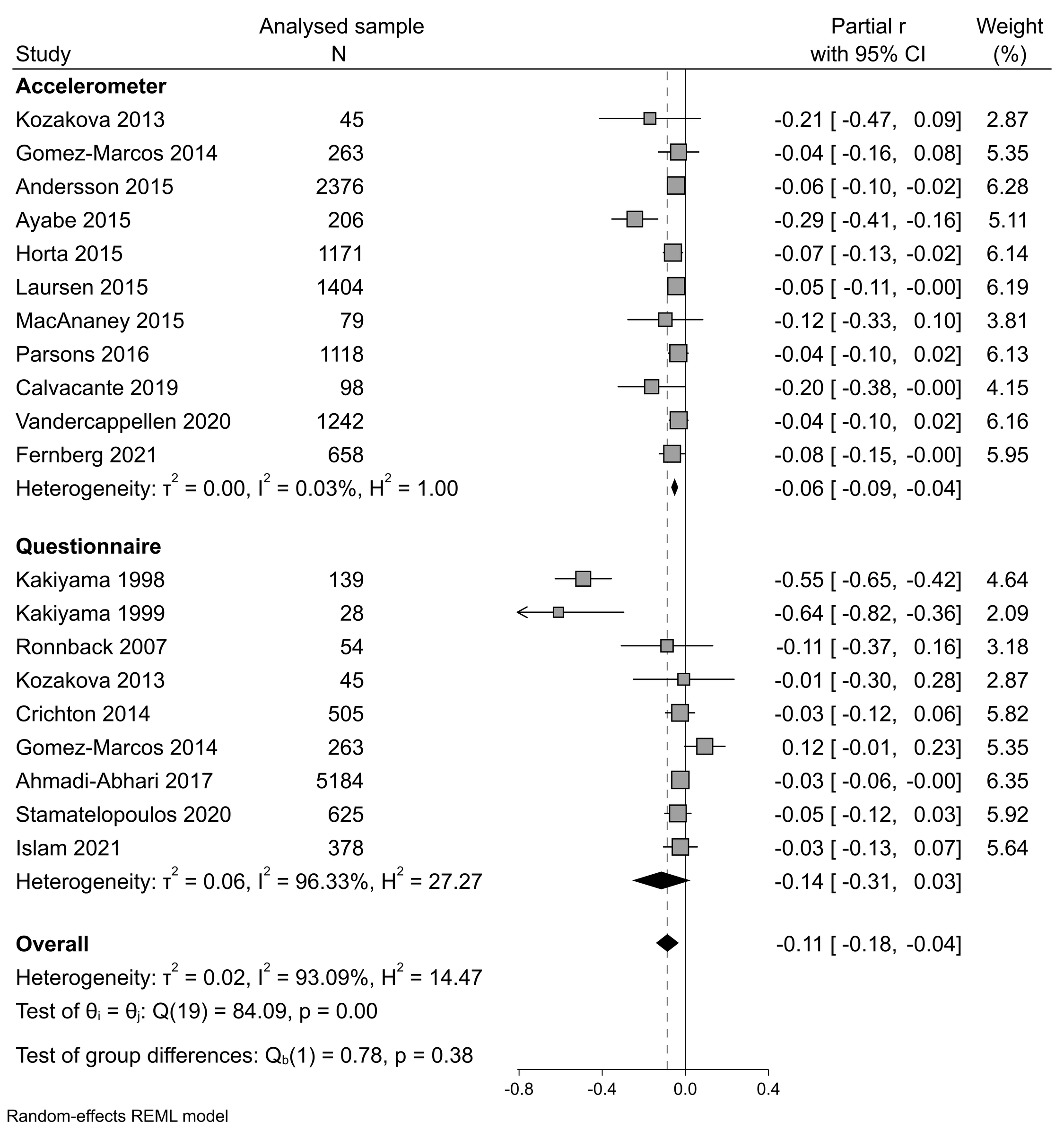

Supplement: S6 Fig — Random effects model. Grey squares indicate individual study association with 95% confidence intervals (CI). Solid vertical line represents line of no difference. Dashed vertical line and black diamond represents the overall summary estimate of association. (TIF) [file pone.0284164.s007.tif]
