# Supplementary material for: Associations of habitual physical activity and carotid-femoral pulse wave velocity; a systematic review and meta-analysis of observational studies
Source: PLoS One. 2023 Apr 6;18(4):e0284164. doi: 10.1371/journal.pone.0284164 (PMC10079053; doi:10.1371/journal.pone.0284164)
Supplement: S1 Table — (PDF) [file pone.0284164.s008.pdf]

S1 Table. Full data extraction summary for all 29 included studies.

Author	Study Type	Country / Cohort	Sample size Male/Female Age (Mean ±SD)	Physical Activity Measure	cfPWV Measure "Device Name" (Method)	Standard Covariates	Study Quality	Main Results	↓ ↔
**Kakiyama et al., 1998**	Cross Sectional	Japan	n=139<br>Male Only<br>39.5 ± 13.5	Stanford Seven-Day Recall Interview	"PWV-200" (Oscillometry)	N	POOR	Total weekly PAEE (kcal/week) r=-0.547, P<0.0001	↓
**Kakiyama et al., 1999**<br>(Study included blind participants. Sighted participant results presented in this review only.)	Cross Sectional	Not Specified	n=40<br>Male Only<br>20.75 ± 1.7	Stanford Seven-Day Recall Interview	"PWV-200" (Oscillometry)	N	POOR	Total weekly PAEE (kcal/week) r=-0.643, P<0.0001	↓
**Perkins et al., 2006**	Cross Sectional	Not Specified	n=115<br>Male Only<br>50.8 ± 7.1	Modified Harvard Alumni Questionnaire	"Complior" (Tonometry)	N	POOR	MVPA (hr/week); R^2=0.006 P=0.401	↔
**Ronnback et al., 2007**<br>(Prospective study design, but only enough information to include cross-sectional analyses.)	Cross Sectional	Helsinki, Finland	n=54<br>Male Only<br>58 (52-78)	Kuiopio Ischaemic heart disease study 12-month PA questionnaire	"Sphygmocor" (Tonometry)	N	FAIR	Total leisure time PA MET-hr/week r=-0.11 (NS)	↔
**Duren et al., 2008**	Cross Sectional	Not Specified	n=26<br>12/14<br>50.4 ± 7	Baecke Habitual Physical Activity Questionnaire	"Tonometry"	Y	POOR	Physical activity score R^2=0.658, P<0.001, PA t=-5.28, P<0.001	↓
**Gando et al., 2010**	Cross Sectional	Not Specified	n=538<br>172/366<br>48.4 ± 9.97	Actimarker Accelerometer	"Form PWV/ABI" (Tonometry)	N	POOR	Total Sample LPA min/day: (N/S)<br>Total Sample: MPA min/day r=-0.14 P<0.01.<br>Total sample VPA min/day r=-0.009 P<0.05<br>Young: LPA (N/S)<br>Young MPA (N/S)<br>Young VPA (N/S)<br>Young Low LPA vs High LPA (split by median) (NS)<br>Middle Aged LPA: (N/S)<br>Middle Aged MPA: r=-0.21 P<0.01. Std. β=-0.22<br>Middle Aged VPA: r=-0.12 P<0.05<br>Middle age Low LPA vs High LPA(NS)<br>Older LPA: r=-0.39 P<0.01. Std. β=-0.39<br>Older MPA: r=-0.31 P<0.01. Std. β=-0.30<br>Older VPA: (N/S)<br>Older Low LPA vs High LPA P<0.05	↔<br>↓<br>↓<br>↔<br>↔<br>↔<br>↔<br>↔<br>↓<br>↓<br>↔<br>↓<br>↓<br>↔<br>↓
**Kozakova et al., 2013**	Cross Sectional	Not Specified	n=45<br>23/22<br>42 ± 9	Uniaxial Accelerometer Model AM7164<br><br>IPAQ	"Complior" (Tonometry)	N<br><br>N	POOR<br><br>POOR	Average counts/min r=-0.21 (NS)<br><br>Total PA MET-min/week r=0.01 (NS)	↔<br><br>↔
**Crichton et al., 2014**<br>(PA measured 4.68 years prior to PWV)	Prospective	New York, USA Maine Syracuse Longitudinal Study Cohort	n=505<br>201/304<br>61.0 ± 11.6	Nurses Health Study Activity Questionnaire	"Sphygmocor" (Tonometry)	NS	POOR	Total baseline MET-hr/week std. β=-0.032 P=0.413	↔

**Gomez-Marcos et al., 2014**	Cross-Sectional	Salamanca, Spain EVIDENT Study Cohort	n=263<br>107/156<br>55.85 ± 12.21	Actigraph GT3X Accelerometer<br><br>PA Recall Questionnaire	"Sphygmocor" (Tonometry)	Y<br><br>Y	GOOD<br><br>GOOD	Counts/min (non-std) B=0.00 [-0.001,0.001] P=0.51<br>LPA min/day B=0.00 [-0.002,0.0012] P=0.86<br>MPA min/day B=0.003 [-0.004,0.000] P=0.43<br>VPA min/day B=0.009 [-0.009,0.027] P=0.32<br><br>MET-hr/week B=0.007 [0.000, 0.015] P=0.06	↔<br>↔<br>↔<br>↔<br><br>↔
**Andersson et al., 2015**	Cross Sectional	England Framingham Heart Study, Third Generation Cohort	n=2376<br>1096/1280<br>47 ± 9	Actical Accelerometer	"Cardiovascular Engineering Inc." (Tonometry)	y	GOOD	Per 10min total (bouts or non-bouts) MVPA/day B=-0.53±0.19 ms/m P=0.006.<br>Per 10min bout MVPA/day B=-0.80±0.30 ms/m P<0.01<br>Per 10min non-bout MVPA/day B=-0.45±0.29 ms/m P>0.05<br>Meeting guidelines (bouts or non-bouts) B=-2.05 ± 0.78ms/m P=0.008<br>Meeting guidelines (>10min bouts) B=-1.82 ± 1.10ms/m P>0.05<br>LPA per 10 min/day B=-0.13 ± 0.09 (NS)	↓<br>↓<br>↔<br>↓<br>↔<br>↔
**Ayabe et al., 2015**	Cross Sectional	Japan Nakanojo Study Cohort	n=206<br>97/109<br>72.8 ± 4.6	Lifecorder Accelerometer	"BP-203RPE" (Oscillometry)	Y	FAIR	MVPA min/day CRUDE: r=-0.264 P<0.001. ADJUSTED: r=-0.295 P=0.004 Multiple regression analysis: r^2=0.251 P<0.001	↓
**Horta et al., 2015**	Cross Sectional	Brazil 1982 Pelotas Birth Cohort	n=1241<br>633/608<br>30 ± 0	GENEActiv Accelerometer	"Sphygmocor" (Tonometry)	Y	FAIR	MVPA (min/day) Quartiles; Q2vsQ1 B=-0.02 95%CI[-0.20, 0.15] Q3vsQ1 B=-0.25 95%CI[-0.42, -0.07] Q4vsQ1 B=-0.29 95%CI[-0.46, -0.11]. P=0.001<br>Raw acceleration (average mg) Quartiles; Q2vsQ1 B=-0.06 95%CI[-0.24, 0.13] Q3vsQ1 B=-0.24 95%CI[-0.42, -0.06] Q4vsQ1 B=-0.37 95%CI[-0.56, -0.19]. P<0.001<br>Continuous MVPA min/day with covariates: Std. β = -0.0744675 SE=0.0010575. P=0.015. Without covariates Std. β=-0.0751507, SE=0.0009712 P=0.008.<br>Split into Inactive (0 min MVPA), Slightly active (<30 min MVPA/day) and Meeting guidelines (≥30 min MVPA/day). Guideline vs Inactive β=-0.35 95%CI[-0.56, -0.14]	↓<br>↓<br>↓<br>↓
**Laursen et al., 2015**	Cross Sectional	Denmark ADDITION-Pro Study Cohort	n=1816<br>934/882<br>66.2	Actiheart combined accelerometer and heart rate monitor.	"Sphygmocor" (Tonometry)	Y	GOOD	10kJ/kg higher daily PAEE Model 1: = 1.2% decrease in cfPWV 95%CI[-1.9, -0.5] Model 3: This decreases to 0.75% difference 95%CI[-1.47, -0.03].	↓
**Mac Ananey et al., 2015**	Cross Sectional	Dublin, Ireland	n=79<br>51/28<br>39 ± 9	Triaxial Accelerometer (RT3)	"Skidmore Medical Vicorder" (Oscillometry)	N	POOR	Relative MVPA min/day r=-0.12 P>0.05<br>LPA min/day r=-0.23 P>0.05<br>MPA min/day r=0.00 P>0.05<br>VPA min/day r=-0.18 P>0.05	↔<br>↔<br>↔<br>↔
**Funck et al., 2016**<br>(Study included T2DM presented here are healthy controls only.)	Cross Sectional	Aarhus, Denmark	n=65<br>36/29<br>58 ± 9.4	Actiheart combined accelerometer and heart rate monitor.	"Sphygmocor" (Tonometry)	Y	FAIR	Low activity group (< median 31counts/min)=9.0+-1.4m/s vs High activity group (> median 31counts/min)=7.7+-1.4m/s P<0.01. After adjustment difference in cfPWV between high and low activity groups = 0.62m/s P=0.06	↔
**Johansson et al., 2016**	Cross Sectional	Finland	n=99<br>Male Only<br>74.3 ± 5.16	Questionnaire / Interview. Not Specified.	"Sphygmocor" (Tonometry)	N	POOR	Total leisure time PA in MET-hr/week T1 (<5.9 MET-hr/week) =10.8±3.7 m/s, T2 (5.9-16 MET-	↔

								hr/week)=10.3±3.4 m/s, T3 (16.1-50MET-hr/week) =9.5±1.8m/s p=0.28<br>Average PA intensity category. 1MET PWV=14.0(3.9), 4MET=10.3(3.2), 8-10MET=9.3(1.8) P<0.001	↓
**Parsons et al., 2016**	Cross Sectional	Britain British Regional Heart Study	n=1118<br>Male Only<br>78.4 ± 4.6	GT3X Actigraph Accelerometer	"Skidmore Medical Vicorder" (Oscillometry)	Y	GOOD	Per 10min MVPA per day B=-0.022m/s 95%CI[-0.054, 0.010]. Std. β= -0.0424 ± 3.2781. Without covariates std β=-0.0952 ± 3.2715.<br>Per 30min LPA per day B=-0.053m/s 95%CI[-0.103, -0.002]<br>Per 10,000 vertical counts per day B=-0.012 [-0.023, -0.002]	↓<br>↓<br>↓
**Ahmadi-Abhari et al., 2017**<br>(PWV & PA measured at baseline, PWV measured 5 years later. Change in PWV shown here.)	Prospective	London, UK Whitehall II cohort	n=5184<br>3797/1387<br>65 ± 5.8	Modified Minnesota PA Questionnaire	"Sphygmocor" (Tonometry)	Y	GOOD	**MVPA hr/week; Model 1;** B= -0.028 m/s smaller increase in cfPWV over 5 years [-0.046, -0.011] P<0.05 compared to the average PWV increase over 5 years = 0.76 m/s. **Model 2:** B=-0.018 [-0.035, -0.0001] P<0.05.<br>**MET-hr/week; Model 1:** B= -0.002 m/s smaller increase in PWV over 5 years 95%CI[-0.004, 0.0004] (N/S) compared to the average PWV increase over 5 years = 0.76 m/s. **Model 2:** B=-0.001 [-0.003, 0.001] (N/S)<br>**Mild PA hr/week; Model 1:** B=0.006 [-0.002, 0.014]. **Model 2:** B=0.006 [-0.002, 0.015].	↓<br>↔<br>↓
**Ahmadi-Abhari et al., 2017**<br>(Baseline Measures)	Cross-Sectional			Modified Minnesota PA Questionnaire	"Sphygmocor" (Tonometry)	Y	GOOD	**Per 1hr/week MVPA, Model 1:** Mean difference in PWV compared to baseline mean (8.4±2.4m/s)= -0.049 [-0.065, -0.033] P<0.05. **Model 2:** =-0.019 [-0.035, -0.0004] P<0.05.<br>**Per 1-MET-hr/week, Model 1:** =-0.003 m/s [-0.004, -0.0009] decrease in PWV compared to mean PWV. **Model 2:** =0.0001 [-0.0016, 0.0019] (N/S)<br>**Medium activity (1hr/week - 2.5hr/week) vs low activity (<1hr/week)** mean difference =-0.15 m/s [-0.31, 0.01] **Model 2**: Mean diff=-0.01 [-0.16, 0.15] (NS). **High activity (>2.5hr/week) vs low** mean diff=-0.37 [-0.5, -0.25] P<0.05. **Model 2:** mean diff=-0.13 [-0.25, -0.01] P<0.05.	↓<br>↔<br>↓
**Bohn et al., 2017**<br>(Study included participants with the metabolic syndrome. Healthy controls only included here.)	Cross-Sectional	Porto, Portugal	n=116<br>41/45<br>42 ± 12	Actigraph GT1M Accelerometer	"Sphygmocor" (Tonometry)	N	FAIR	MVPA: Less active (<30min/day)=8.7m/s, More active (>30min/day) = 8.4m/s (N/S)<br>LPA: Less active (<30min/day)=8.5m/s, More active (>30min/day) = 8.6m/s (N/S)	↔<br>↔
**Tanaka et al., 2018**<br>(PA measured at baseline and 6yr, PWV measured 24yrs after baseline)	Prospective	USA ARIC Study Cohort	n=3893<br>1587/2306<br>75.2 ± 5	Baecke Habitual Physical Activity Questionnaire	"Tonometry"	Y	GOOD	Compared to no activity, **Low activity (1st tertile)** cfPWV=-0.18 95% CI[-0.42, 0.07] (N/S), **Moderate activity (2nd tertile)** cfPWV=-0.30 95% CI[-0.55, -0.05] P<0.05, **High activity (3rd tertile)** cfPWV=-0.38 95%CI[-0.63, -0.12] P<0.05.	↓
**Tanaka et al., 2018**<br>(Analysis only including subsample whose PA remained the same over 6yrs)	Prospective		n=1747<br>665/1082<br>74.9 ± 4.9		"Tonometry"			Compared to persistently no activity, persistently Low activity (1st tertile) cfPWV=-0.18 95% CI[-0.58, 0.22] (N/S), Moderate activity (2nd tertile) cfPWV=-0.21 95% CI[-0.62, 0.21] (N/s), High activity (3rd tertile) cfPWV=-0.73 95%CI[-1.10, -0.36] P<0.05.	↓
**Calvacante et al., 2019**	Cross-Sectional	Porto, Portugal	n=98<br>Male Only	Actigraph GT1M Accelerometer	"Sphygmocor" (Tonometry)	Y	FAIR	LPA (min/day) r=-0.24 p<0.05, adjusted for age r=0.25 p<0.01. β=-0.23 P=0.004	↓

			55.3 ± 7.31			N		MVPA (min/day) r=-0.24, p<0.05. β=-0.20 P=0.015<br>Meeting Guidelines cfPWV = 9.76 ± 1.52 m/s vs not meeting guidelines cfPWV = 10.53 ± 1.79 m/s, p<0.05	↓<br>↓
**Deiseroth et al., 2019**<br>(Interventional study including "at risk" of CVD group - Cross-sectional baseline for Healthy participants only included here)	Cross-Sectional	Basel, Switzerland<br>EXAMIN-AGE Study Cohort	n=68<br>27/41<br>59.5 ± 7	Frieburg Short Form PA Questionnaire	"Sphygmocor" (Tonometry)	Y	GOOD	Active (>9METs/week) vs Sedentary (≤ 3METs/week)<br>**Model 1:** adjusted R^2=0.34 mean difference = 0.64m/s 95% CI[0.07, 1.20] P=0.027. **Model 2;** adjusted R^2=0.38 mean difference = 0.63m/s 95% CI[0.08, 1.18] P=0.026	↓
**Hamaguchi et al., 2020**	Cross-Sectional	Japan	n=22<br>Female Only<br>55.1 ± 6.2	Active Style Pro HJA-350IT Triaxial Accelerometer	"Form PWV/ABI" (Tonometry)	N	POOR	Low PWV group PA (MET-hr) =5.7(2.6), High PWV group PA=4.6(2.1), P=0.30	↔
**Stamatelopoulos et al., 2020**	Cross-Sectional	Athens, Greece	n=625<br>Female Only<br>57.7 ± 7.6	Short Form International Physical Activity Questionnaire	"Complior" (Tonometry)	N	POOR	Low PA r=0.085.<br>Moderate PA r=-0.082.(NS)<br>Vigorous PA r=-0.083. (NS)<br>Total PA r=-0.045 (NS).	↔<br>↔<br>↔<br>↔
**Vandercappellen et al., 2020**<br>(Study included participants with DM, healthy controls only included here.)	Cross-Sectional	Netherlands<br>Maastricht Study Cohort	n=1242<br>537/705<br>59.57 ± 7.8	Activ-PAL3 Accelerometer	"Sphygmocor" (Tonometry)	Y	FAIR	Total PA hr/week std. β=-0.03 (-0.08, 0.01) P=0.139<br><br>Insufficiently active vs inactive= -0.11 [-1.10, 0.14], weekend warrior vs inactive = -0.22 [-0.5, 0.05], regularly active vs inactive = -0.3[-0.56, -0.03) P<0.05.	↔<br><br>↓
**Yee Lee et al., 2020**	Cross-Sectional	Singapore<br>ELIXA Cohort Study	n=80<br>43/37<br>42.25 ± 15.78	Self-declared five-year history of exercise participation including light intensity activities.	"Sphygmocor" (Tonometry)	Y	POOR	Compared to 0min/week PA, <180min/week, 180-360, and >360min/week PA (N/S) (no actual values)	↔
**Fernberg et al., 2021**	Cross-Sectional	Sweden	n=658<br>180/478<br>22 ± 2	ActiGraph GT3X+ Accelerometer	"Sphygmocor" (Tonometry)	Y	FAIR	**WOMEN:** LPA (min/day) B=-0.126, std β=-0.038 (NS)<br>MVPA (min/day) B=-0.148, std β=-0.104 (NS)<br>Total PA (min/day) B=-0.260, std β=-0.076 (NS)<br>**MEN:** LPA (min/day) B=-0.300, std β=-0.089 (NS)<br>MVPA (min/day) B=0.011, std β=0.007 (NS)<br>Total PA (min/day) B=-0.009, std β=-0.088 (NS)<br>No significant differences in PWV were seen between the groups, <30min, 30min, 60min (data not shown)	↔<br>↔<br>↔<br>↔<br>↔<br>↔<br>↔
**Nosrati-Oskouie et al., 2021**	Cross-Sectional	Mashhad, Iran	n=658<br>321/337<br>41.8 ± 6.3	Physical Activity Questionnaire designed by Persian Cohort.	"Sphygmocor" (Tonometry)	N	POOR	PA groups (above/below median PA (38.6 METs/hour/day)) B=-0.015 (-0.039 to 0.008) std β=-0.051 P=0.206	↔
**Islam et al., 2021**	Cross-Sectional	Georgia, USA	n=378<br>150/228<br>52.8 ± 10.3	Jackson Heart Study (JHS) Physical Activity Questionnaire	"Sphygmocor" (Tonometry)	Y	FAIR	Total PA Score B=−0.03 (−0.14, 0.07) P= 0.56<br>Active Living Index B=0.15 (−0.13, 0.43) P=0.30<br>Work Index B=−0.08 (−0.51, 0.34) P=0.70<br>Home/life Index B=−0.32 (−0.63, −0.02) P=0.04<br>Sport/exercise Index B=−0.60 (−1.14, −0.06) P=0.03<br>Total Physical Activity Score Q1 vs Q4 Model 2: B=0.16 (−0.41, 0.73) P= 0.58<br>Active Living Index Q1 vs Q4 Model 2: B=0.38 (−0.17, 0.92) P=0.18<br>Work Index Q1 vs Q4 Model 2: B=0.01 (−0.78, 0.80) P=0.98<br>Home/life Index Q1 vs Q4 Model 2: B=−0.63 (−1.17, −0.08) P=0.02	↔<br>↔<br>↔<br>↓<br>↓<br>↔<br>↔<br>↔<br>↓

	Sport/exercise Index Q1 vs Q4 Model 2: B=−0.64 (−1.19, −0.10) P=0.02	↓

cfPWV Measure; “Name of device used” (tonometry or oscillometric method of measurement)

Standard Covariates Y/N; Standard = age, sex, body mass index and blood pressure.

↓↔**;** indicates direction of result; ↓ significant negative association**.** ↔ No association.

PA; physical activity. LPA; Light intensity physical activity. MPA; Moderate intensity physical activity. VPA; Vigorous physical activity. MET; Metabolic equivalents. PAEE; physical activity energy expenditure. cfPWV; carotid femoral pulse wave velocity.

Age; represented as mean age ± standard deviation or Median (inter-quartile range).
