# Supplementary material for: Associations of habitual physical activity and carotid-femoral pulse wave velocity; a systematic review and meta-analysis of observational studies
Source: PLoS One. 2023 Apr 6;18(4):e0284164. doi: 10.1371/journal.pone.0284164 (PMC10079053; doi:10.1371/journal.pone.0284164)
Supplement: Attachment — Submitted filename: Response to Reviewers.docx [file pone.0284164.s009.docx]

PONE-D-22-16539

Associations of habitual physical activity and carotid-femoral pulse wave velocity; a systematic review and meta-analysis of observational studies.

**We thank the editor and reviewer for taking the time to read our manuscript and for their useful and constructive comments. Please find our responses below.**

1. Please ensure that your manuscript meets PLOS ONE's style requirements, including those for file naming. The PLOS ONE style templates can be found at

[https://journals.plos.org/plosone/s/file?id=wjVg/PLOSOne_formatting_sample_main_body.pdf](https://nam12.safelinks.protection.outlook.com/?url=https%3A%2F%2Fjournals.plos.org%2Fplosone%2Fs%2Ffile%3Fid%3DwjVg%2FPLOSOne_formatting_sample_main_body.pdf&data=05%7C01%7Crl442%40exeter.ac.uk%7Cd22de2691e864a45589708da96ab549b%7C912a5d77fb984eeeaf321334d8f04a53%7C0%7C0%7C637987959846177444%7CUnknown%7CTWFpbGZsb3d8eyJWIjoiMC4wLjAwMDAiLCJQIjoiV2luMzIiLCJBTiI6Ik1haWwiLCJXVCI6Mn0%3D%7C2000%7C%7C%7C&sdata=u9GHKFpB8OxM5fb5OR4LZWOLpg%2Brd2IYM6XMr0F6qW8%3D&reserved=0)  and

[https://journals.plos.org/plosone/s/file?id=ba62/PLOSOne_formatting_sample_title_authors_affiliations.pdf](https://nam12.safelinks.protection.outlook.com/?url=https%3A%2F%2Fjournals.plos.org%2Fplosone%2Fs%2Ffile%3Fid%3Dba62%2FPLOSOne_formatting_sample_title_authors_affiliations.pdf&data=05%7C01%7Crl442%40exeter.ac.uk%7Cd22de2691e864a45589708da96ab549b%7C912a5d77fb984eeeaf321334d8f04a53%7C0%7C0%7C637987959846177444%7CUnknown%7CTWFpbGZsb3d8eyJWIjoiMC4wLjAwMDAiLCJQIjoiV2luMzIiLCJBTiI6Ik1haWwiLCJXVCI6Mn0%3D%7C2000%7C%7C%7C&sdata=3sjaySPnyx7CHvivsI15a%2FN9GO0WYuLFPUGWtV0NfLc%3D&reserved=0)

**Author response: Thank you. We have been through the PLoS ONE style requirements and have made edits to ensure it meets the required standard.**

2. We note that you have included the phrase “data not shown” in your manuscript. Unfortunately, this does not meet our data sharing requirements. PLOS does not permit references to inaccessible data. We require that authors provide all relevant data within the paper, Supporting Information files, or in an acceptable, public repository. Please add a citation to support this phrase or upload the data that corresponds with these findings to a stable repository (such as Figshare or Dryad) and provide and URLs, DOIs, or accession numbers that may be used to access these data. Or, if the data are not a core part of the research being presented in your study, we ask that you remove the phrase that refers to these data.

**Author Response: No data has been removed, rather 3 new supplementary files have now been added to the manuscript.**

3. Please include captions for your Supporting Information files at the end of your manuscript, and update any in-text citations to match accordingly. Please see our Supporting Information guidelines for more information: [http://journals.plos.org/plosone/s/supporting-information](https://nam12.safelinks.protection.outlook.com/?url=http%3A%2F%2Fjournals.plos.org%2Fplosone%2Fs%2Fsupporting-information&data=05%7C01%7Crl442%40exeter.ac.uk%7Cd22de2691e864a45589708da96ab549b%7C912a5d77fb984eeeaf321334d8f04a53%7C0%7C0%7C637987959846177444%7CUnknown%7CTWFpbGZsb3d8eyJWIjoiMC4wLjAwMDAiLCJQIjoiV2luMzIiLCJBTiI6Ik1haWwiLCJXVCI6Mn0%3D%7C2000%7C%7C%7C&sdata=Z30oDtpT4%2Bs8kSP3eFfea5wQ80xFSFckXVtqV%2BV96so%3D&reserved=0)

**Author Response: We have now added a “supporting information” section at end of manuscript with associated captions.**

**Comments to the Author**

5. Review Comments to the Author

Please use the space provided to explain your answers to the questions above. You may also include additional comments for the author, including concerns about dual publication, research ethics, or publication ethics. (Please upload your review as an attachment if it exceeds 20,000 characters)

Reviewer #1: The introduction begins with information about the problem of cardiovascular disease (CVD), especially its impact on the public health system. Then the protective effects of exercise, recommendations, and the inverse relationship between CVD and exercise. The third paragraph showed information about other cardiovascular risk factors but that do not alter the inverse relationship between exercise and CVD. However, the sequence of ideas in the fourth and fifth paragraph is confusing. Information about vascular health appears, then about exercise and then the different methods of vascular outcome measurement. It would be interesting to organize the ideas in paragraphs 4 and 5 for a better understanding of the study's problem.

**Author response: Thank you for this comment, we welcome the opportunity to clarify this section. To improve the flow of the latter part of the introduction and clarify the rationale for the review we have made a number of edits and have moved the discussion of vascular function and its assessment to earlier in the piece. This is now followed by discussion of evidence for associations between structured exercise and vascular function, and the evidence gaps which this review addresses, namely the lack of clarity regarding associations between habitual physical activity and arterial stiffness and the limitations of existing reviews.**

In line 165 “Authors were contacted to obtain this information if not reported in the paper (N=14)”, Does the number refer to the authors contacted or to the number of information not obtained?

**Author response: Thank you, we appreciate the opportunity to clarify. The text has now been edited to read: “We contacted the corresponding authors of 14 studies to request any information required for meta-analysis that was not reported in their papers."**

**The resultant information not obtained from contacting Authors and therefore necessitating manual conversion is then detailed in the following bullet points 1-3, together with citations of the individual studies.**

In line 176. “In eight studies30 33-40 univariate” the number of the references does not correspond to the information described.

**Author response: Thank-you for pointing out this error. These references have been checked and the extra reference (30) removed.**

In line 206. “Five studies34 44-46 included a group” revise the number of references.

**Author response: Thank-you for pointing out this error. There were only four studies included a subset of participants with disease. These are specified correctly. The text has been edited to describe the “four” rather than ‘five’ studies.**

In line 214. 16 measured hPA via self-report questionnaire or interview.27 30 34-36 38-40 42 43 46 48 50 56-58.

**Author Response: These references have been double checked and are indeed correct. No changes have been made.**

In figure 1, if we consider all the excluded reports from the reports assessed for eligibility, 13 studies remain. Probably one study fell into more than one exclusion criterion. It would be clearer to describe this information in the results.

**Author response: This is correct, we appreciate the opportunity to clarify. Several studies were excluded based on them meeting more than one exclusion criteria. This has been addressed by the addition of “(not mutually exclusive)” within figure 1 for clarity.**

**Additionally, this information has now been added in full in lines 206-212 (clean manuscript file, or lines 218-225 in track changes manuscript file) as follows: “Database searches yielded 8149 studies of which 180 full-texts were screened for eligibility. 151 studies were excluded for the following reasons: Investigating clinical population without healthy control group (N=21), Measuring exercise only with no measurement of habitual PA (N=50), alternative measurement of vascular function without cfPWV (N=51), insufficient data reported (N=37) and the inadequate categorization of participants restricting analysis (N=8). Note, a number of studies were excluded for meeting more than one of these exclusion criteria. A PRISMA flow diagram is presented in Fig 1.”**
