# Supplementary material for: Associations of habitual physical activity and carotid-femoral pulse wave velocity; a systematic review and meta-analysis of observational studies
Source: PLoS One. 2023 Apr 6;18(4):e0284164. doi: 10.1371/journal.pone.0284164 (PMC10079053; doi:10.1371/journal.pone.0284164)
Supplement: Attachment — Submitted filename: response to reviewers.docx [file pone.0284164.s010.docx]

PONE-D-22-16539

Associations of habitual physical activity and carotid-femoral pulse wave velocity; a systematic review and meta-analysis of observational studies.

**We thank the editor and reviewer for taking the time to read our manuscript and for their useful and constructive comments. Please find our responses below.**

1) The purpose of this present review is to clarify the associations between habitual physical activity and arterial stiffness, responding to the limits of a previous review. This information appears in the introduction and there is nothing further. It would be interesting to have an approach on both studies, the previous review and the current one in the discussion.

**Author Response: Thank you for this comment, we agree that it is useful to compare the results from the present review with that of the previous review, on which we are providing an update. The text in the first paragraph of the discussion has now been amended to compare the results from our own pooled analyses with that of the previous review.**

2) If I understand correctly, data adjusted for age, sex, BMI and BP were used. However, the units of measurement of physical activity are extremely variable. In the authors' opinion, is it possible that the association between habitual physical activity and PWV was not evident from these differences? Perhaps trying to classify the studies according to the amount of physical activity and/or intensity would reveal a stronger association. Explore more the information of the lines 388-393.

**Author Response: Thank you for your comment. We agree that the measurement units of habitual physical activity (hPA) were very variable within included studies. It is for this reason that we converted all association results to partial r or standardised beta – by doing this we are instead reporting the association between per standard deviation change in hPA, rather than in absolute units (of which are variable). This allowed us to make direct comparisons across different studies that have measured and reported hPA in different ways.**

**The amount of physical activity and the intensity of physical activity was considered when completing our analyses. A subgroup analysis was completed on studies reporting associations with total PA and compared to those reporting associations with MVPA (figure 3 in the manuscript). However unfortunately it was not possible to complete separate pooled analysis for associations with light intensity activity due to the limited number of studies reporting this association (N=6) and the lack of data available from these six studies meaning it was not possible to convert the available association statistics to partial r / standardised beta for comparison across studies.**

**Similarly, we decided it would not be appropriate to complete a subgroup analysis, or meta-regression on the average level of PA completed by the cohort within each study due to the limited number of included studies that reported average PA level (N=9 out of 18) and the differing units of PA measurement reported within these 9 studies.**

3) In the discussion between the lines the authors cite physical training. The comparison cited between the results of this review and previous studies of physical training needs to be approached with caution. First, because of the limitations cited above regarding self-reporting of physical activity. Second, because this is not the initial purpose of this review. Finally, because the results of this review are not the effects of interventional studies of physical training. However, exploring why the results are consistent across studies despite differences in modalities may be interesting and may help answer my previous question.

**Author Response: We thank you for your comment and agree it is not useful to compare the results from the present review to those of previous meta-analyses on exercise training interventions. This text has now been edited to remove reference of training intervention studies and instead provide a detailed comparison with that of the previous systematic review on habitual PA and cfPWV.**
